# Targeting glutamate uptake to treat alcohol use disorders

**DOI:** 10.3389/fnins.2015.00144

**Published:** 2015-04-23

**Authors:** P.S.S. Rao, Richard L. Bell, Eric A. Engleman, Youssef Sari

**Affiliations:** ^1^Department of Pharmacology, College of Pharmacy and Pharmaceutical Sciences, University of ToledoToledo, OH, USA; ^2^Department of Psychiatry, Indiana University School of MedicineIndianapolis, IN, USA

**Keywords:** glutamate, GLT1, EAAT2, dopamine, alcohol, neurotransmitter

## Abstract

Alcoholism is a serious public health concern that is characterized by the development of tolerance to alcohol's effects, increased consumption, loss of control over drinking and the development of physical dependence. This cycle is often times punctuated by periods of abstinence, craving and relapse. The development of tolerance and the expression of withdrawal effects, which manifest as dependence, have been to a great extent attributed to neuroadaptations within the mesocorticolimbic and extended amygdala systems. Alcohol affects various neurotransmitter systems in the brain including the adrenergic, cholinergic, dopaminergic, GABAergic, glutamatergic, peptidergic, and serotonergic systems. Due to the myriad of neurotransmitter and neuromodulator systems affected by alcohol, the efficacies of current pharmacotherapies targeting alcohol dependence are limited. Importantly, research findings of changes in glutamatergic neurotransmission induced by alcohol self- or experimenter-administration have resulted in a focus on therapies targeting glutamatergic receptors and normalization of glutamatergic neurotransmission. Glutamatergic receptors implicated in the effects of ethanol include the ionotropic glutamate receptors (AMPA, Kainate, and NMDA) and some metabotropic glutamate receptors. Regarding glutamatergic homeostasis, ceftriaxone, MS-153, and GPI-1046, which upregulate glutamate transporter 1 (GLT1) expression in mesocorticolimbic brain regions, reduce alcohol intake in genetic animal models of alcoholism. Given the hyperglutamatergic/hyperexcitable state of the central nervous system induced by chronic alcohol abuse and withdrawal, the evidence thus far indicates that a restoration of glutamatergic concentrations and activity within the mesocorticolimbic system and extended amygdala as well as multiple memory systems holds great promise for the treatment of alcohol dependence.

## Introduction

A direct link has been established between chronic alcohol consumption and at least 50 different diseases and disorders (Reed et al., 1996; Rehm et al., 2003). Moreover, alcohol use disorders (AUDs) are the third leading cause of preventable death, affecting over 18 million adults and resulting in over 100,000 deaths in the U.S. annually (Grant et al., 2004; Mokdad et al., 2004; Johnson, 2010). Economically, AUDs and their associated effects cost society approximately 200 billion dollars or more each year (Harwood et al., 1999). The pattern of drinking and total volume consumed per unit time are important characteristics for diagnosing AUDs and for developing treatment strategies as well as determining epidemiological antecedents and disease trajectory (Heather et al., 1993; Lancaster, 1994; Zucker, 1995; Shield et al., 2013). These characterizations have led to classifications, typologies and/or drinking profiles for alcoholics (Cloninger, 1987; Babor et al., 1992; Epstein et al., 1995; Lesch and Walter, 1996; Conrod et al., 2000). In addition, it appears that the pharmacological efficacy of some treatments for AUDs is influenced by the phenotypic and/or genotypic characteristics associated with these typologies (Epstein et al., 1995; Johnson et al., 2003; Cherpitel et al., 2004; Dundon et al., 2004; Johnson, 2004, 2010).

The fact that people from similar environments often differ in their pattern and frequency of alcohol use as well as the well-documented familial incidence of alcoholism underscores the substantial role of genetics in the development and expression of AUDs (Cotton, 1979; Cloninger, 1987; Enoch and Goldman, 2001).

**Figure 1:**
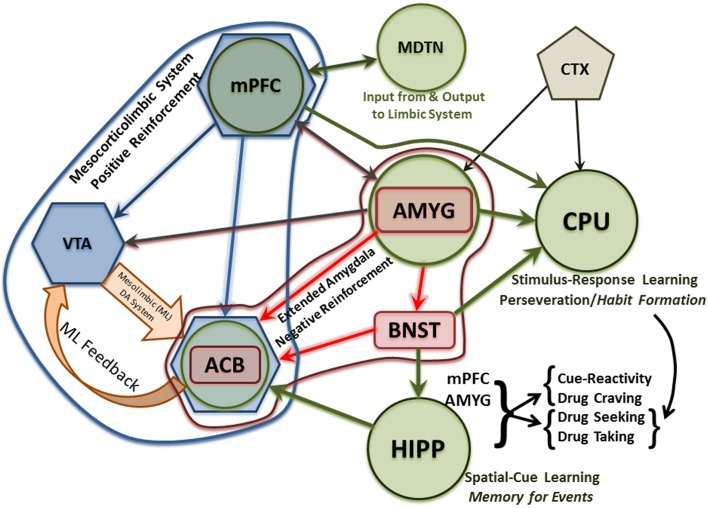
**Diagram of glutamatergic neurocircuits within the mesocorticolimbic system (blue hexagonals and associated arrows), extended amygdala (red rectangles and associated arrows) as well as related memory (green circles and associated arrows) and limbic structures involved in the development of alcohol/drug dependence**. The early experimentation/intoxication stage of alcohol/drug abuse is marked by neuroplastic changes in the mesocorticolimbic system. Later, the extended amygdala plays an increasingly greater role, relative to the mesocorticolimbic system (although these systems interact as seen in this Figure), in mediating continued alcohol/drug usage despite physical, emotional, social and/or economic consequences. In later stages of alcohol dependence, most of the neuroplastic changes to the mesocorticolimbic and extended amygdala systems attributed to repeated cycles of alcohol/drug abuse—withdrawal—and relapse have already occurred. While associative learning, contextual conditioning, and habit formation have been occurring throughout the addiction cycle, highlighted are these multiple memory systems (that interact and often overlap) which experience a substantial increase in glutamatergic activity-mediated neuroplastic changes. Basic descriptors of the memory processes mediated by the hippocampus and caudate-putamen, as well as the stages of relapse they influence, are also indicated. ACB, Nucleus Accumbens; AMYG, Amygdala; BNST, Bed Nucleus of the Stria Terminalis; CPU, Caudate Putamen; CTX, Cortex; HIPP, Hippocampus; MDTN, Medial Dorsal Thalamic Nucleus; ML, Mesolimbic; mPFC, Medial Prefrontal Cortex; VTA, Ventral Tegmental Area.

In general, AUDs define a chronic, progressive, relapsing disorder that advances in stages from experimentation to dependence (Volkow and Li, 2005; Heilig and Egli, 2006; Koob and Le Moal, 2008; Koob, 2009; Spanagel, 2009; Jupp and Lawrence, 2010; Koob and Volkow, 2010). The disease progresses from rewarding, euphoric and positive-reinforcement aspects (e.g., motor and autonomic activation as well as facilitating pro-social behavior) that drive the disease-process in the early stages to the dysphoric and associated negative-reinforcement aspects (e.g., removal of withdrawal-associated effects) of chronic alcohol use that drive the process in later stages. And, while the end-point is the same, the progression for each individual is often different and nonlinear in nature (i.e., individuals will return to earlier stages of the disease cycle with varying frequency and intensity). The change in primacy from positive reinforcement to negative reinforcement across the addiction cycle follows neuroplastic changes in central reward neurocircuitry (e.g., the mesocorticolimbic and extended amygdala systems) induced by chronic alcohol abuse. In the normal functioning brain, a balance (i.e., homeostasis) exists between excitatory and inhibitory neurotransmission. Acute alcohol consumption disturbs this equilibrium by enhancing inhibitory and attenuating excitatory neurotransmission (e.g., Roberto et al., 2003; Basavarajappa et al., 2008; Leriche et al., 2008). However, following long term alcohol consumption, the brain compensates for the depressant effects of alcohol to maintain homeostasis between inhibitory (e.g., GABA) and excitatory (e.g., glutamate) neurotransmission by increasing excitatory activity and reducing inhibitory activity (e.g., Nam et al., 2012; Koob, 2013; Tabakoff and Hoffman, 2013). These changes occur in a number of brain regions within the mesocorticolimbic and extended amygdala reward circuits. And, these brain regions display heavy glutamatergic innervation as shown in Figure 1, which describes some of these glutamatergic projections and is adapted from a number of studies (Aston-Jones et al., 2009; Bonci and Borgland, 2009; Carlezon and Thomas, 2009; Haydon et al., 2009; Kalivas et al., 2009; O'dell, 2009; Renthal and Nestler, 2009; Russo et al., 2009; Taylor et al., 2009; Vezina and Leyton, 2009; Volkow et al., 2009, 2010; Wheeler and Carelli, 2009; Kenny, 2011; Luscher and Malenka, 2011; Potenza et al., 2011; Sulzer, 2011; Kash, 2012; Bass et al., 2013; Chambers, 2013; Cui et al., 2013; Jennings et al., 2013; Volkow and Baler, 2013). In this review, we have focused on alcohol-induced changes in glutamate uptake, the role of glutamate transporters in the development of alcohol dependence, and current and promising glutamate-associated pharmacotherapies for the treatment of alcohol dependence.

## The mesocorticolimbic system, extended amygdala and glutamatergic neurotransmission

The primary glutamatergic projections, associated with addiction include the mesocorticolimbic dopamine system and the extended amygdala as well brain regions mediating learning and memory (Figure 1). The mesocorticolimbic system is a primary neurocircuit mediating reward and reinforcement salience that is most often, but not always, associated with a positive valence (Tzschentke, 2000; Wise, 2005; Robinson and Berridge, 2008; Brooks and Berns, 2013; Volman et al., 2013). Regarding the dual valence-processing of rewarding/reinforcing stimuli within the mesocorticolimbic system and extended amygdala, it has been shown that activation of the glutamatergic projections from the amygdala through the bed nucleus of the stria terminalis to the ventral tegmental area (VTA) results in aversive and anxiogenic behaviors, whereas activation of the GABAergic parallel projections results in appetitive and anxiolytic behaviors (Jennings et al., 2013). Interestingly, this dissociation between glutamatergic activity and aversion vs. GABAergic activity and reward is also true for similar parallel projections from the VTA to the lateral habenula (Stamatakis et al., 2013; Root et al., 2014). Therefore, from a purely neurochemical perspective within these neurocircuits, it is not surprising that the use of ethanol during the experimentation and early stages of abuse is positively reinforcing and rewarding, given that the acute actions of ethanol are to inhibit glutamatergic activity and potentiate GABAergic activity. During early stages of drug/alcohol abuse positive reinforcement drives continued usage, such that the individual consumes or self-administers the drug or alcohol for the pleasant and euphoric effects they induce. Positive reinforcement is mediated, for the most part, by the mesocorticolimbic dopamine system, which is controlled to a great extent by glutamatergic activity. Similarly, given that the chronic actions of ethanol are to inhibit GABAergic activity and potentiate glutamatergic activity, and a consequent increase in dopaminergic activity within the VTA, accumbens (acb), and prefrontal cortex (PFC), it results in an aversive state especially under conditions of withdrawal. It also recruits increased glutamatergic and corticotrophin releasing factor anxiogenic activity in the amygdala/extended amygdala. Combined, these latter effects elicit continued alcohol and drug usage in an attempt to delay/block/reverse the onset of withdrawal/aversion-associated symptoms.

Early stages of alcohol/drug dependence are also marked by increased glutamatergic activity within the extended amygdala and the presence of neuroplastic changes in the mesocorticolimbic system. Dependence is associated with the development of tolerance and attendant decreases, as a result of neuroplastic changes, in positive reinforcement (e.g., mesocorticolimbic dopaminergic activity). Dependence is also associated with withdrawal, which is correlated with a number of symptoms linked to increased glutamatergic/excitatory neuronal activity and decreased GABAergic/inhibitory neuronal activity. The extended amygdala mediates, at least in part, withdrawal symptoms related to anxiety and irritability as well as accompanying heightened physiological and autonomic responses. These heightened responses are mediated not only by increased glutamatergic (as opposed to GABAergic) activity but also increased anxiogenic peptidergic activity [i.e., corticotrophin releasing factor (CRF) as opposed to anxiolytic neuropeptide Y (NPY) activity] within the extended amygdala, particularly the AMYG and BNST (Roberto et al., 2012). However, progression through these stages of the addiction cycle is not linear in nature. Rather, there are substantial individual differences in the number and duration of instances where an individual's usage pattern returns to that seen in a previous stage of the addiction cycle.

During late stages of dependence, the neuroplastic changes in the mesocorticolimbic system are retained and the neuroplastic changes of the extended amygdala become more pronounced. As highlighted in Figure 1, multiple memory systems (See White, 1996, White, and associated commentaries, for an early review) are also recruited in these neurocircuits with substantial glutamatergic activity. Neuroplastic changes within different memory systems strengthen the behavioral manifestations seen in late stage dependence. Given that alcohol and drug addiction are often characterized as compulsive behaviors, it has been repeatedly postulated, albeit with different terminology, that the caudate-putamen, which mediates habit formation and memory, tends to predominate as a memory circuit in late stage dependence (Koob and Volkow, 2010; Volkow et al., 2010; Everitt and Robbins, 2013; Richard et al., 2013; Zorrilla and Koob, 2013; Wise and Koob, 2014).

Prominent among these behaviors in later stages of dependence is the ubiquitous phenomenon of relapse (Chiauzzi, 1991; Weiss et al., 2001; Barrick and Connors, 2002; Jaffe, 2002). Relapse takes place during withdrawal, which distinguishes it from continued usage in the absence of withdrawal. Relapse is often precipitated by some form of cue-reactivity, which would require contextual processing by the hippocampus as well as autonomic activity modulated by the amygdala and medial PFC. This autonomic/physiological activity can be modulated by all of the brain regions ascribed, in the Figure 1, to the mesocorticolimbic system and extended amygdala. The desire to remove associated aversive symptoms/stimuli, or to re-live memories of a previous positive experience, with the former tending to predominate over the latter, leads to drug/alcohol seeking and taking with the recruitment of both habit- and cognitive-memory circuits, again with the former predominating over the latter in late stage dependence.

## Glutamatergic neurotransmission: interactions with alcohol

The major excitatory central neurotransmitter, glutamate, acts on two broad categories of receptors, ionotropic and metabotropic. The ionotropic receptors are ligand-gated ion channels and are further classified into N-methyl-D-aspartate (NMDA), alpha-amino-3-hydroxy-5-methylisoxazole-4-propionic acid (AMPA) and Kainate receptors based on their sensitivity to respective agonists (Nakanishi, 1992). The metabotropic receptors (mGluR) are G-protein coupled receptors divided into eight mGluR subtypes, having multiple splice variants (Pin and Duvoisin, 1995). As indicated in Section The Mesocorticolimbic System, Extended Amygdala and Glutamatergic Neurotransmission, ethanol exposure alters the central glutamatergic activity and these changes vary depending on the extent of ethanol abuse.

Chronic ethanol exposure (Rossetti and Carboni, 1995; Dahchour and De Witte, 1999, 2000; Melendez et al., 2005; Kapasova and Szumlinski, 2008; Ding et al., 2012), for instance, has been shown to elevate extracellular levels of glutamate in mesocorticolimbic brain areas. Moreover, the effects of ethanol consumption on NMDA- and mGlu-R as well as Homer (a glutamatergic receptor scaffolding protein) subunit expression in the extended amygdala and Acb core are also influenced by the length of ethanol-withdrawal (Obara et al., 2009). Intracellularly, ethanol inhibits, non-competitively, the NMDAR-mediated calcium influx in the cortex, Acb, amygdala, hippocampus, and VTA (Nie et al., 1994; Wirkner et al., 2000; Roberto et al., 2004; Stobbs et al., 2004; Zhu et al., 2007). Additionally, ethanol's effects on NMDA receptor activity are followed by phosphorylation and internalization of NR2 subunits (Suvarna et al., 2005). Ethanol was also found to inhibit NMDA-induced increases in cyclic guanosine monophosphate activity (Hoffman et al., 1989). Furthermore, studies have shown that alcohol withdrawal is associated with increased excitatory amino acid transmission, leading to manifestation of symptoms such as seizures, which can be blocked using NMDA receptor antagonists (Danysz et al., 1992; Snell et al., 1996; Malinowska et al., 1999; Narita et al., 2000; Bienkowski et al., 2001; Nelson et al., 2005).

Glutamate activity at mGluR5, a post-synaptic metabotropic glutamate receptor, has been shown to regulate self-administration of alcohol in rats (Besheer et al., 2008). Similarly, antagonists of mGluR5 are also found to be beneficial in preventing alcohol relapse in a genetic animal model of alcoholism (Schroeder et al., 2005). Thus, pharmacological blockade or very low levels of *mGluR2* mRNA is associated with increased alcohol consumption by alcohol preferring as well as alcohol non-preferring rats (Li et al., 2010; Holmes et al., 2013; Meinhardt et al., 2013; Zhou et al., 2013). Similarly, post-mortem analysis of human frontal cortex from alcohol-dependent patients revealed a decreased expression levels of mGluR2 mRNA (Meinhardt et al., 2013) further highlighting its involvement in the development and/or expression of alcohol dependence.

## Glutamate transporters: role in glutamate homeostasis

Glutamate transporters are membrane-bound protein pumps found on both neuronal and glial membranes; these transporters regulate glutamate uptake. Increased extracellular glutamate may cause calcium homeostasis dysfunction, increased nitric oxide production, activation of proteases, increased cytotoxic transcription factor levels, and increased free radicals that may subsequently lead to neuronal death (For review, see Wang and Qin, 2010). There are two types of glutamate transporters that regulate extracellular glutamate level: sodium dependent excitatory amino acid transporters (EAATs) and vesicular glutamate transporters (VGLUTs). In addition, there exists a cysteine-glutamate antiporter that regulates the exchange of cysteine and glutamate at the synapse (Bridges et al., 2012).

### Excitatory amino acid transporters

EAATs are present in both presynaptic neurons and glial cells and are responsible for modulating glutamate homeostasis (Kanai et al., 1995; Danbolt, 2001). Glutamate is co-transported along with three sodium ions and one proton followed by efflux of one potassium ion (Zerangue and Kavanaugh, 1996). Five subtypes of EAATs have been identified in human and rodent brains. The first three subtypes that were identified in rat/rabbit models are called glutamate/aspartate transporter (GLAST) (Storck et al., 1992), GLT1 (Pines et al., 1992), and excitatory amino acid carrier type 1 (EAAC1) (Kanai and Hediger, 1992); their human counterparts are termed EAAT1, EAAT2, and EAAT3, respectively (Arriza et al., 1994). The other two subtypes were identified in both rodents and humans and named as EAAT4 (Fairman et al., 1995) and EAAT5 (Arriza et al., 1997). The five EAAT subtypes have been found to share 50–60% sequence homology (Seal and Amara, 1999). The localization of these five EAAT subtypes has been studied in detail and described elsewhere (Gegelashvili and Schousboe, 1998; Danbolt, 2001).

### Vesicular glutamate transporters

VGLUTs are responsible for the uptake and sequestration of glutamate into presynaptic vesicles for storage. The uptake is driven by a proton-dependent electrochemical gradient that exists across the vesicle membrane and depends on the potential gradient created by a vacuolar-type ATPase (Edwards, 2007). Three isoforms of VGLUTs have been identified in the mammalian central nervous system (CNS): VGLUT1, VGLUT2, and VGLUT3 (El Mestikawy et al., 2011). These transporters belong to the type 1 phosphate transporter family. VGLUT1 is expressed in the cortex, hippocampus, and thalamus while VGLUT2 is found in the neocortex, olfactory bulb, dentate gyrus, and subiculum; also, co-expression of VGLUT1 and VGLUT2 has been observed in the hippocampus (Herzog et al., 2006). VGLUT1 is suggested to be expressed at synapses associated with low release rates and long-term potentiation, while VGLUT2 is expressed at synapses with high release rates and long-term potentiation (Fremeau et al., 2001), with others suggesting VGLUT2 is also associated with synapses displaying long-term depression (Kaneko and Fujiyama, 2002). Alternatively, VGLUT3 was identified in neuronal somato-dendrites and glia distributed sparsely throughout the brain. The expression of VGLUTs is believed to be on the extra-presynaptic terminal, suggesting a probable role in modulation of glutamate signaling via endocytosis (Fremeau et al., 2002), although some work has suggested a role for VGLUTs in glutamatergic exocytosis as well (Bellocchio et al., 1998; Fremeau et al., 2004; Seal and Edwards, 2006).

### Cysteine–glutamate antiporter

Cysteine–glutamate antiporter is a plasma membrane-bound, Na^+^-independent, anionic amino acid transporter that exchanges extracellular cysteine for intracellular glutamate and serves as a source of non-vesicular glutamate release (Danbolt, 2001). It exists as two separate proteins: the light chain cysteine/glutamate exchanger (xCT) that is unique to the cysteine-glutamate antiporter, and the heavy chain 4F2 that is common to many amino acid transporters (Sato et al., 1999). Similar to EAATs, this antiporter is distributed on cells throughout the body and preferentially on glia in the brain (Bridges et al., 2012). Furthermore, it provides tone to mGluRs and cysteine for glutathione synthesis, thereby antagonizing oxidative stress. Given glutamate's role in excitotoxicity, it is not surprising that cysteine-glutamate exchange is thought to play a prominent role in regulating extracellular glutamate levels (Murphy et al., 1989; Bridges et al., 2012).

Studies have shown that a reduction in the cysteine/glutamate exchange increased susceptibility to relapse-seeking behavior (Baker et al., 2003). Also, restoration of the antiporter activity through intracranial perfusion of cysteine or systemic administration of N-acetyl cysteine was shown to decrease cocaine seeking in rat models (Baker et al., 2003). Another study showed that ceftriaxone treatment restored both GLT1 and xCT levels, which in turn inhibited relapse to cocaine-seeking behavior (Sari et al., 2009; Knackstedt et al., 2010). Similarly, we have recently demonstrated chronic alcohol consumption in male alcohol-preferring P rats (a genetic animal model of alcoholism) to be associated with a significant decrease in expression of xCT in Acb and PFC (Alhaddad et al., 2014a). Overall, the cysteine-glutamate antiporter is known to play an important role in maintaining glutamate homeostasis under normal and various pathological conditions (For review, see Bridges et al., 2012).

### Role of glutamate reuptake in alcohol dependence

Glutamate-mediated excitotoxicity is implicated in trauma, ischemia and several other neurodegenerative disorders (Sattler and Tymianski, 2001). Glial sodium dependent transporters, GLAST (EAAT1) and GLT1 (EAAT2), in particular GLT1, are responsible for at least 90% of extracellular glutamate removal (For review, see Anderson and Swanson, 2000). Impaired glutamate uptake due to dysfunction or downregulation of EAAT2 results in several neurological disorders, including Amyotrophic Lateral Sclerosis (ALS), Alzheimer's disease, epilepsy, ischemia and hepatic encephalopathy (Maragakis and Rothstein, 2006). Importantly, we have previously demonstrated that chronic exposure to alcohol results in significant down-regulation of GLT1 expression in the Acb and/or PFC in P rats (Sari and Sreemantula, 2012; Sari et al., 2013).

Furthermore, studies have demonstrated a significant reduction in the levels of EAAT1 and EAAT2 in the basolateral amygdala in postmortem human alcoholic brains as compared to non-alcoholic individuals (Kryger and Wilce, 2010). Acute exposure to alcohol has been found to inhibit glutamatergic neurotransmission. However, chronic exposure to alcohol may elevate glutamate levels, which can lead to withdrawal-associated effects of alcohol deprivation and exposure subject's seeking alcohol-associated negative reinforcement to remove the same (Valenzuela, 1997). Additionally, a reduction in the expression of GLAST level and an increase in GLAST mRNA were found in post-mortem human PFC samples of alcoholics, which might be due to a compensatory mechanism induced by chronic alcohol abuse/dependence (Flatscher-Bader and Wilce, 2008). Furthermore, an increase in VGLUT2 expression in the Acb shell was observed, while VGLUT1 remained unchanged, following chronic alcohol exposure and subsequent deprivation from alcohol (Zhou et al., 2006).

Importantly, GLT1 is currently considered a molecular target for the attenuation of alcohol dependence since it regulates the majority of extracellular glutamate uptake (Rao and Sari, 2012). Previous studies have identified several beta-lactam antibiotics as potent modulators (i.e., elevation) of GLT1 expression (Rothstein et al., 2005). Based on its favorable pharmacokinetic properties, ceftriaxone (a third-generation cephalosporin) was chosen for further study in *in vitro* models of ischemic injury and motoneuron degeneration and *in vivo* animal models of ALS (Rothstein et al., 2005). Furthermore, screening of several FDA-approved compounds using a luciferase reporter assay on human astroglial cells identified harmine, a beta-carboline alkaloid, as a potent EAAT2 promoter. Further testing in cell cultures and ALS animal models demonstrated that harmine effectively increased both GLT1 protein and glutamate transporter activity (Li et al., 2011). Thus, harmine may prove efficacious in the treatment of alcohol dependence.

## Current pharmacotherapy targeting glutamate neurotransmission for alcohol dependence

Current available therapeutics for alcohol dependence target several different systems due to the non-specific nature of alcohol's action in the brain. In general, the available FDA-approved therapies for alcohol dependence work by blunting the rewarding effects (naltrexone) of ethanol, creating an aversion (disulfiram) toward ethanol or restoring the homeostatic balance between inhibitory and excitatory neurotransmission in the CNS (acamprosate). Ideally, drugs targeting the mesocorticolimbic reward pathway and counteracting chronic ethanol exposure-induced adaptations would present a feasible pharmacological solution.

Acamprosate is a synthetic GABA analog, FDA-approved drug for the prevention of alcohol relapse. It was suggested to be a functional NMDA antagonist, thereby countering chronic alcohol exposure-induced increases in glutamate concentrations and the subsequent precipitation of a hyperglutamatergic state, which occur during alcohol withdrawal episodes (Dahchour et al., 1998). Acamprosate treatment has also been shown to affect GABAergic neurotransmission by inhibiting presynaptic GABA_B_ receptors (Berton et al., 1998). Studies have suggested that acamprosate treatment is mildly effective or ineffective in multi-center clinical trials (Anton et al., 2006; Mason et al., 2006). However, further analysis has suggested that, apart from being a well-tolerated drug, acamprosate treatment is associated with significant positive effects on rates of abstinence among both sexes (Mason and Lehert, 2012).

Topiramate, an FDA approved anti-epileptic drug, has shown promising results in attenuating alcohol consumption due to its modulation of glutamatergic neurotransmission. Topiramate has been demonstrated to inhibit the AMPA and kainate glutamate receptors (Skradski and White, 2000; Gryder and Rogawski, 2003). While topiramate treatment was associated with decreased ethanol intake in several animal studies (Hargreaves and Mcgregor, 2007; Nguyen et al., 2007; Breslin et al., 2010), the drug treatment was not associated with changes in ethanol conditioned place preference (Gremel et al., 2006). In addition, topiramate has been shown to be effective in reducing alcohol intake and preventing relapse in human subjects (Baltieri et al., 2008; Florez et al., 2008). Other anticonvulsants [e.g., gabapentin (another synthetic GABA analog altering GABAergic, glutamatergic and adrenergic activity with some efficacy for treating cannabis dependence; Howland, 2013) or valproate] have also been evaluated; with a meta-analysis suggesting anticonvulsants, which generally modulate glutamatergic, GABAergic and possibly other excitatory or inhibitory neuronal activity, have modest but significant efficacy in the treatment of alcohol dependence (Pani et al., 2014).

## GLT1 upregulators: potential targets for the treatment of alcohol dependence

### Ceftriaxone

Ceftriaxone is a third-generation, semi-synthetic cephalosporin with a broad spectrum of activity against Gram-positive and Gram-negative aerobic, and a few anaerobic, bacteria. Ceftriaxone can be administered either intravenously or intramuscularly and has been successfully used in the treatment of meningitis; urinary tract infections; lower respiratory tract infections; skin, soft tissue, bone, and joint infections; and bacteremia/septicemia (Richards et al., 1984). In addition to its GLT1 modulating effect (Rothstein et al., 2005), ceftriaxone has been studied extensively for its neuroprotective activity. In an animal model of stroke, ceftriaxone treatment was associated with a significant reduction in acute stroke mortality and improved neurological performance (Thone-Reineke et al., 2008). Additionally, a ceftriaxone-mediated increase in GLT1 expression in the spinal cord was found to be beneficial in treating opioid-induced paradoxical pain and neuropathic pain (Ramos et al., 2010). Ceftriaxone has been studied extensively for its potential role in treating ethanol dependence (for review see Rao and Sari, 2012). We have previously reported that ceftriaxone treatment administered intraperitoneally (i.p.) in P rats attenuated ethanol consumption during five consecutive daily doses of the treatment (Sari et al., 2011). Importantly, attenuation of ethanol consumption following ceftriaxone treatment was associated with upregulation of GLT1 expression levels in the mesocorticolimbic pathway, including the Acb and PFC. Additionally, ceftriaxone administered i.p. was found effective in attenuating ethanol withdrawal and relapse-like drinking behaviors (Qrunfleh et al., 2013; Abulseoud et al., 2014). Furthermore, apart from modulating GLT1 expression, we have demonstrated upregulation of xCT expression as an additional mechanism of ceftriaxone-induced normalization of glutamatergic homeostasis in the Acb and PFC (Alhaddad et al., 2014a; Rao and Sari, 2014b). Ceftriaxone was also effective in attenuating ethanol consumption in P rats subjected to a long-term ethanol drinking paradigm (Rao and Sari, 2014a). Furthermore, our recent findings have demonstrated that ceftriaxone-induced GLT1 upregulation is associated with activation of AKT and NF-kB signaling pathways in the Acb and PFC of P rats (Rao et al., 2015).

### GPI-1046

GPI-1046, 3-(3-pyridyl)-1-propyl (2S)-1-(3,3-dimethyl-1,2-dioxopentyl)-2-pyrrolidinedinecarboxylate, is a synthetic non-immunosuppressive ligand of FK506 binding protein-12 (Steiner et al., 1997b). FK506 was found to promote neuronal survival. Thus, a series of non-immune suppressive ligands of FKBP-12 were synthesized, including GPI-1046, the prototype for these non-immunosuppressive ligands. GPI-1046 was shown to be effective in neurodegenerative animal models (Steiner et al., 1997a). Along with neuroprotective properties, GPI-1046 was also found to possess anti-retroviral activity and was effective in inhibiting HIV replication (Steiner et al., 2007). Interestingly, GPI-1046 was found to have an upregulatory effect on GLT1 expression both *in vitro* and *in vivo* (Ganel et al., 2006). Importantly, we recently reported that GPI-1046 treatment (i.p.) upregulated GLT1 protein expression in the PFC and Acb and concomitantly reduced alcohol intake in male P rats (Sari and Sreemantula, 2012). Further, studies are warranted to reveal the molecular mechanism involved in GPI-1046 mediated changes in GLT1 expression.

### MS-153

We have recently reported that a synthetic compound, (R)-(–)-5-methyl-1-nicotinoyl-2-pyrazoline (MS-153) reduced alcohol intake in male P rats (Alhaddad et al., 2014b). This reduction in alcohol intake was associated in part with upregulation of GLT1 expression in the Acb. This compound, in addition to GLT1 upregulation, was shown to activate p-Akt and NF-kB pathways; these signaling pathways were previously suggested to be involved in GLT1 upregulation. In addition, MS-153 mediated changes in GLT1 expression were also examined in the amygdala and hippocampus of P rats (Aal-Aaboda et al., 2015). While ethanol consumption significantly down-regulated expression of xCT and GLT1, MS-153 treatment normalized expression of these glutamate transporters in both the amygdala and hippocampus of P rats compared to vehicle control animals.

## Conclusion and closing thoughts

Alcohol consumption affects a number of neurotransmitters and neuromodulators in the CNS, including dopamine, serotonin and GABA. Pertinent to the present paper, the neurotransmitter glutamate has been shown to be critical in the development and expression of alcohol dependence. In particular, changes in glutamatergic activity are often associated with neuroplastic changes in CNS circuitry (Bliss et al., 2014). These changes occur in brain structures mediating reward, reinforcement, learning and memory (for reviews on some of these changes see White, 1996). In general, based on the existing literature, chronic alcohol and other drugs of abuse result in a pronounced increase in glutamatergic activity within these neuronal circuits. Therefore, any pharmacological treatments that can reverse this hyperglutamatergic state hold great promise in the treatment of alcohol, and possibly illicit drug, dependence. It is noteworthy that compounds that physically and/or functionally upregulate glutamate transporters, in particular GLT1, are ideal candidates for further exploration.

### Conflict of interest statement

The authors declare that the research was conducted in the absence of any commercial or financial relationships that could be construed as a potential conflict of interest.
